# History of the Pathology of Tuberculous Pulmonary Phthisis, from Laennec to Koch

**Published:** 1897-10

**Authors:** Q. C. Smith

**Affiliations:** 617 Colorado St.; Austin, Texas


For the Texas Medical Journal.

### HISTORY OF THE PATHOLOGY OF TUBERCULOUS PULMONARY PHTHISIS, FROM LAENNEC TO KOCH.

BY Q. C. SMITH, M. D., AUSTIN, TEXAS.

[Read before Travis County Medical Society, September 2d, 1897.]

LAENNEC, was the father of the present era, in the history of pathology of tuberculous pulmonary phthisis; hence we begin our brief and imperfect review, with the discoveries and teachings of the great inventor of the stethoscope.

One of the main objects of this paper is, to utilize the history of the pathology of tuberculous pulmonary phthisis, during recent times, to show the folly and perniciousness of beliefs, opinions, and practices, founded upon *theory*. For, it is as true to-day, as when Plato taught, that "*theories* stink of ignorance." Laennec, was the originator of the idea, and the first to bring out definitely and clearly, the specific nature and unity of the pathology of tuberculous phthisis pulmonalis. He believed, that tubercle, is an accidental product, foreign to the normal state, having no analogue in the sound body.

The insight of his genius, enabled him to discern an identity of origin amid the apparent diversity of form and appearance, denominated military tubercle, grey granulations, tuberculous infiltrations; grey or yellow, are all of the same nature and origin.

Laennec, also maintained, that tubercle is not only a pathological substance, foreign to the normal organism, but that its evolutionary manifestations prove it to be a specific and *suigeneris* substance. And just here, it will be profitable for us to note and remember, that the reason that these (then) new and peculiar views of Laennec, being founded upon numerous well digested *facts*, as carefully and laboriously observed by himself, (and not upon *theory*) have triumphed over all attacks, both by his contemporaries and later opponents. Prominent among the former was Broussais, and later, Niemeyer, the latter leading what was then termed the "new German school," following Broussais, said, that inflamatory action, was the *fons et origo* of all forms of tuberculous disease: And that phthisis, resulted from caseous degeneration of the products of simple inflammation; that when in a phthisical lung the grey granulations and pneumonia co-existed, the granulations were secondary and consecutive; and so arose Niemeyer's famous dictum, that "The greatest danger to which a phthisical patient could be exposed, is, that he should become tubercular."

As a result of the teachings of Broussais, and the "new German school," many lost faith in the correctness of Laennec's teaching, aud fell in with the later idea, of the several varieties of phthisis, resulting from the various modifications, variety and stages of inflammatory processes. This *theory* of Niemeyer, founded upon shallow reasoning, taking effect for cause, yet so plausible, plain, and seemingly self-explanatory, was a rescue haven for ignorance, and dogmatic incompetency, for cliniciens, as well as for pathologists, which is utilized even to this day.

But the inflammatory *theory* of Broussais, and later, of Niemeyer, resting as it did on a "creation of the human mind," and not upon *facts*, could not remain "established."

While Laennec's teachings, founded upon well digested *facts*, have stood the test of progressive enlightment, and numerous critical investigators, and to-day stands vindicated and definitely confirmed by the most brilliant conquests of modern technique.

Louis, was the only great cotemporary that believed with Laennec: while Andral, Cruveibheir, Bouillaud, and many others, held with Broussais, and later, with Niemeyer, that tubercle was a result of inflammatory processes; and therefore non-specific.

The two essential points in the doctrine of Broussais, and the "new German school," as to the pathological nature of tubercular pulmonary phthisis, were irritation the *cause*, and a lymphatic product the *effect*, have held quite prominent places in the history of the pathology of phthisis.

In 1848, Reinhardt, vigorously attacked Laennec's views, and with much learning and plausible theorizing, endeavored to prove that Laennec's views were erroneous. And later, about 1850, Virshow, coincided with Reinhardt, and declared, that most of the anatomical appearances recognized by the Laennec as tuberculous, were merely inspissated pus, together with epithelial cells, filling the pulmonary alveoli, and were the results of inflammatory action attendant upon catarrhal pneumonia.

And Virchow, taught, that pulmonary phthisis, might be due, either to the evolution of tubercle, or to caseous hepatization; and that phthisis properly so-called, was rather the result of caseous hepatization, than of tubercle. He reserved the name "tuberculous," to acute military tuberculosis. And held, that Laennec's idea, of the *unity* of phthisis, and that it is always a tuberculous disease, is erroneous. Vichow, then taught,—and so teaches yet,—that the greater part of pulmonary phthisis, is due to imflammatory action.

Thus we see, that for Laennec, and his followers, there is but one kind of pulmonary phthisis, i. e. tuberculous; and for Virchow, Niemeyer, and their followers, there are at least two; one a caseous pneumonia, and the other, a rarer form, i. e. the tuberculous. The duality doctrine, led by Virchow, Niemeyer, and many other eminent pathologists, obtained wide credence and for many years, general acceptance, especially outside of France.

But in France, the most distinguished pathologists and clinicians, in latter years, rallied to the standard of Laennec; and later still, under the able leadership of the astute pathologist and clinicien, Grancher, Laennec's views were diffused and popularized even down to recent years. Grancher's thorough and profound researches, in reference to the correctness of Laennec's teaching, enabled him to formulate a pathological law; that "a nodule of caseous pneumonia, has the same structure, as the typical tuberculous granulation." And upon critical investigation of the evidence Grancher produced to support aforesaid law, Rindfleisch, and later Charcot, united with many other distinguished investigators, in support of Laennec's teachings.

To speak very briefly, this hurried glance, over this large and industriously tilled field, brings us down to the period of about 1860.

During the decade from 1860 to 1870, Villemin, of Paris, originated and carried out a series of experiments, that enabled him to discover, what is to-day, considered the true *fons et origo* of tubercular phthisis: he, thus becoming the true and original discoverer of the parasitic origin of tubercular phthisis, instead of Robt. Koch: the sensational character of whose announcements, in reference to this subject, created such great commotion, and therapeutic disappointment, in 1882. And notwithstanding the fact, that an important and influential commission of, and from, the French Academy of Sciences, after long, careful, deliberate testing of Villemin's experiments, fully confirming the truth of his conclusions, already published; some of his jealous cotemporaries, in various quarters, notably, Wilson Fox and Burdon Sanderson, of London; and Virchow and his followers, in Germany, performed numerous experiments, sacrificing a great number of rodents, seeking evidence to refute the views of Villemin: and, as a matter of course, found what they sought for, and so published with great display of profound erudition. But the true leaven that Villemin had injected into the perverse mass of professional dogmatism, could not be neutralized or destroyed, even by the adverse reports of his distinguished self-sufficient confreres. And this thaumaturgic leaven of Villemin, being the extract derived from carefully observed *facts*, hence the essence of true progress, continued to permeate, energise, and enlighten, the professional mass, until the great Villemin became the pathological father of the famous Robt. Koch.

Surely, this is a remarkable and instructive lesson in the history of pathology: a gruesome field, in which none would be supposed to dig or delve, save in search of the priceless jewel of truth. But Villemin found an able defender in Chauveau,—the great veterinarian,—whose skillful experiments and observations on the transmissibility of tubercle, in oxen and horses, afforded ample proof and corroboration of the conclusions of Villemin. But "truth creeps slowly, whilst error flies," and dies a hard, lingering death. So we observe, so late as 1873, while the foregoing facts were fresh in mind, that at a set discussion of this subject, before the pathological society of London, the *erroneousness* of the teachings of Wilson Fox, Burdon Sanderson, Virchow, and others, were still unrecognized by the leading pathologists of London. And one of their leaders, in said discussion—more teachable than a majority of his fellows,—said: "Fifteen years ago, I came from Germany, strongly impressed with the opinion, that phthisis might be subdivided into many absolutely diverse diseases—diseases diverse in their essential nature, as well as in their apparent origin. I had many terms at the end of my tongue—Broncho-pneumonia, caseous pneumonia, scrofulous pneumonia, etc.,—but when I myself became a teacher, I felt great doubt and difficulty in saying what was *not* tubercle, and still more in saying what *was* tubercle."

But it is needless to follow this conscientious teacher through the long, laborious series of observations, that enabled him to come to the settled conviction of the *unity* of the origin of the morbid processes which constitute tubercular phthisis: as had already been long held and taught, by Laennec, Louis, Villemin, Rindfleisch, Grancher, Cornil, Charcot, Chauveau, and many others.

But the idea of the *unity* of the origin of tubercular phthisis, was not acceptable to the majority of the authorities who took part in aforesaid notable debate. One prominent pathological authority, present at said debate, dogmatically asserted, that "phthisis, was an affection, of which tubercle could no longer be considered as the pathological essence. That the common varieties of pulmonary phthisis, were to be regarded as due almost solely to various forms of chronic inflammatory changes in the lung; tubercle, being merely an occasional and quasi-accidental complication." And he found it difficult to come to the conclusion, that there could be anything *specific*, appertaining to either the pathology or therapeutics of phthisis. It is instructive to remember, that, at that time (1873),—and until the present, in some dark corners—there existed a widespread hostility to the idea, of *anything*, condition or process, being of a *specific* nature, either in pathology or therapeutics.

*Now*, everything is *specific*, from Shamblow's "purely vegetable liver pill," up to Koch's bacillus tuberculosis. Even the veteran consumptologist, C. J. B. Williams, of Brompton Hospital, and father of the present distinguished chief of that institution, believed, that tubercular phthisis pulmonalis, originated in non-specific inflammatory conditions. No wonder he cured such a small per cent. of his patients.

These brief extracts and references, plainly show, the hampering confusion and want of definiteness, that widely obtained less than twenty-five years ago, even in the minds of eminent teachers and pathologists, as to the true pathology of phthisis: and what is of infinitely greater importance, also shows how the slavish following of *theories*—in reference to *any* subject—always, and inevitably leads to erroneous thinking, and pernicious doing.

During the period intervening between the aforesaid discussion (1873), and the date of Robt. Koch's announcement of his discovery, April 10, 1882, much good research work was done, along the lines followed and pointed out by Laennec, Villemin, and Chauveau. Chauveau, by a large and long series of experiments, ascertained, and, by frequent repetition, confirmed the fact, that the infective virus of tubercular phthisis, was often found in the fluids of phthisical cattle, as well as of men, which led him to the conclusion that there were minute particles, imperceptible even under the microscope, inert in appearance, but endowed with tenacious power, and intense virulence.

Buhl, in 1873, and Klebs, in 1875, published their belief, that bacteria are the cause of tubercle, and that they probably existed in the caseous masses. In 1877, Klebs professed to have isolated and cultivated the "*monas tuberculosum*," but he failed to establish its specific character.

In 1881, Toussaint, in France, and Baumgarten, in Germany, and other investigators, discovered microbes, which they thought, might be the active agents in the production of tuberculosis.

In 1881, Prof. Bouchard, of Paris, argued vigorously, on general grounds, that tuberculous phthisis, must be classed amongst the infective diseases. And next year, April 10, 1882, Robt. Koch, announced to the Physiological Society of Berlin, that he had succeeded in isolating and cultivating the microbe of tuberculosis; and it was thus left to a German to give absolute demonstration of the causal *unity* of tubercular phthisis, in all its various forms and manifestations, as had been taught in the early part of the century by the great Frenchman, Laennee.

And this great event, may fitly close our brief historical retrospect of the pathology of tuberculous phthisis pulmonalis, in recent times.

In a brief review of the salient points of this short and imperfect retrospect, as *cliniciens*, nothing stands out so prominently as the momentous warning: that we should not allow *theories* to interfere with or influence our *practice.*

For the experience of all the ages, since the sagacious sage of Cos, sat by the bedside to study therapeutics, rather than from an alchemic cloister, evolve a beautiful "creation of the human mind," has intelligent empiricism, been the father, and directing guide, of all true therapeutic *progress*, and safe clinical *practice.*

Indeed, the pernicious effects that have resulted from permitting *theories*—pathological or otherwise—to govern clinical practice, is the most prominent and mortifying feature in the history of therapeutics.

To briefly illustrate, we will cite only two examples: Following the pathological *theory* of Broussais (already referred to), his disciples practiced free venesection and spare diet, as curative measures, in the clinical treatment of tubercular phthisis pulmonalis. For, said they, "if we remove the fuel (blood), the fire (inflammation) will be extinguished, and thereby the patient's chances for recovery be greatly increased."

And furthermore, said they: "We observe, as has been taught from ancient times, that many of those phthisical patients who lose blood by hemoptysis, are more likely to improve or recover, either with or without treatment, than phthisical patients who do not so bleed: Hence, in the practice of venesection, we are following the plain indications of *vis medicatrix naturæ.*"

The foregoing, is at once, a beautiful piece of sophistry, and a striking illustration of the old adage: "that we find what we *look* for."

While to-day, many of the devoted believers in the *theory* of Koch, reason no more profoundly, or practice more wisely—than did said Broussaians—when they endeavor to so saturate tubercular patients with microbicides, that the tubercle bacilli may either be killed or expelled.

For, say said microphobists: "Has not the distinguished *German* pathologist, Robt. Koch, proved beyond a doubt, that the specific microbe known as *the tubercle bacillus*, to be the indubitable *fons et origo* of tubercular phthisis pulmonalis? And have not we, ourselves, slayed myriads of all sorts of microbes, with various poisonous chemicals, while *finishing* our medical education, in the magnificent new pathological laboratory of Berlin, where constantly abides the august Shekinah of perfected Science, and where the world-renowned microbe killer reigns gloriously supreme?"

Here, again, we see, even the latest so-called "*scientific theory*," leading to *disastrous practice.* The *shallowness* of the therapeutic reasoning, that lead to either and both of aforesaid injurious plans of treatment, is readily apparent to any intelligent clinician. For a knowledge, however thorough and profound of chemistry, pharmacy, pathology, physiology, etiology, diagnosis, nosology, physics, biology and bacteriology, or all these branches of science combined, does not necessarily produce an intelligent therapeutist: as many of our quasi-scientific teachers would have us believe. Howbeit, all the fore-mentioned branches of science, as well as all other sources of useful knowledge, should be put under vassalary tribute, as obedient messengers, each bringing valuable information to the headquarters of the Master Clinician, enabling him to plan his campaigns and battles as best he may, against our common great enemy, Death.

617 Colorado St.

